# Confirmatory methods for endotracheal tube placement in out-of-hospital settings: A systematic review of the literature

**DOI:** 10.1016/j.heliyon.2024.e28479

**Published:** 2024-03-26

**Authors:** Amani Alenazi, Abdullah Alshibani

**Affiliations:** a Barts and the London School of Medicine and Dentistry, Queen Mary University of London, London, United Kingdom; b Emergency Medical Services Department, College of Applied Medical Sciences, King Saud Bin Abdulaziz University for Health Sciences, Riyadh, Saudi Arabia; c King Abdullah International Medical Research Centre, Riyadh, Saudi Arabia

**Keywords:** Endotracheal intubation, Tracheal intubation, Prehospital, Emergency, Ambulance

## Abstract

**Background:**

Confirming proper placement of an endotracheal tube (ETT) is important, as accidental misplacements may occur and lead to critical injuries, potentially leading to adverse outcomes. Multiple methods are available for determining the correct ETT placement in prehospital care.

**Objective:**

To assess the accuracy and reliability of the different methods used to confirm endotracheal intubation in prehospital settings.

**Methods:**

A comprehensive literature search was performed in the MEDLINE, EMBASE, Scopus, and Web of Science databases for studies that were published between 1-June-1992 and 12-June-2022 using a combination of predetermined search terms. Studies that met the inclusion criteria were included and assessed for risk of bias using “Risk of Bias in Non-randomized Studies of Intervention” tool.

**Results:**

Of the 1016 identified studies, nine met the inclusion criteria. Capnography and point-of-care ultrasound showed high sensitivity and specificity rates when applied to confirm ETT placement in prehospital care. Other methods including capnometry, colorimetric detectors, ODDs, and auscultation showed varied sensitivity and specificity. Patient comorbidities and device failure contributed to decreased accuracy rates in prehospital care. Capnography was less reliable in distinguishing between endotracheal intubation and right main stem intubation, which is known as a complication in out-of-hospital endotracheal intubation. Point-of-care ultrasound was more accurate and reliable in detecting oesophageal and endobronchial misplacements. ETCO_2_ monitors, i.e., capnometry and colorimetric detectors, were less reliable in patients with low perfusion states.

**Conclusion:**

This systematic review showed that there is no single method with 100% accuracy in confirming the correct ETT placement and detecting the occurrence of accidental oesophageal or endobronchial misplacements in prehospital care. Further studies with a larger sample size are needed to assess the accuracy of multiple confirmatory methods in prehospital settings.

## List of abbreviations

ETI: Endotracheal Intubation
ETT: Endotracheal Tube
OHCA: Out-of-Hospital Cardiac Arrest
ETCO_2_: End-Tidal Carbon Dioxide
POCUS: Point-of-Care Ultrasound
ODD: Oesophageal Detector Device
PRISMA: “Preferred Reporting Items for Systematic Reviews and Meta-Analyses”
PICO: “Population, Intervention, Comparison, and Outcome”
CPR: Cardiopulmonary Resuscitation
ODB: Oesophageal Detector Bulb
USA: United States of America
CPAP: Continuous Positive Airway Pressure

## 1 Introduction

Multiple challenges are associated with endotracheal intubation (ETI) in prehospital settings. In particular, severe complications can occur when performing ETI in prehospital settings, including hemodynamic collapse, cardiac arrest, tracheal aspiration, airway oedema, bleeding, and multiple intubation attempts due to misplacement of the Endotracheal Tube (ETT) either into the right bronchus or oesophagus [1,2]. For instance, Wirtz et al. reported that among prehospital intubation attempts, 11 out of 132 (8.3%) intubations were incorrectly placed in the oesophagus, and only one (9.1%) of these patients survived till hospital discharge [3]. In an observational study, Silvestri et al. found that without using continuous end-tidal carbon dioxide (ETCO_2_) monitoring, the rate of endotracheal misplacement was around 23% in prehospital settings; however, it was reduced to almost zero after employing continuous ETCO_2_ monitoring [4]. Recently, there are a variety of different methods for confirming ETT placement, including capnography, capnometry, colorimetric ETCO_2_ detector, ultrasound (US), auscultation, and the use of an oesophageal detector device (ODD), in addition to clinical assessments, such as directly visualizing if the ETT passes through the vocal cords into the trachea. However, no method has been reported to be 100% effective and without any limitations in confirming ETI and determining any accidental misplacements. Thus, it is usually recommended to employ several methods for confirming tube position such as the triad of confirmation, which includes capnography/carbon dioxide confirmation, auscultation, and verifying whether the tube passes through the vocal cord.

It is hoped that additional insight into the effectiveness of various strategies for endotracheal tube confirmation will contribute to enhance the quality of prehospital care provided for patients requiring emergency care. Therefore, this review was focused on assessing the accuracy and reliability of applying such confirmation methods.

## 2 Methods

This systematic review was conducted in accordance with the “Preferred Reporting Items for Systematic Reviews and Meta-Analyses (PRISMA)” checklist for systematic reviews [5]. The primary outcome of this review was to report the accuracy of the different methods used to confirm ETT placement in prehospital care in terms of sensitivity and specificity or the positive and negative predictive values of the method. The secondary outcomes were to compare the time taken by each method to confirm ETT position in prehospital care and identify any limitations associated with the use of any confirmation method.

### 2.1 Search strategy and eligibility criteria

A systematic literature search was performed in four databases: MEDLINE, EMBASE, Scopus, and Web of Science. A list of search terms and MeSH keywords were generated and combined by “AND” or “OR” to identify and review the most pertinent literature. The search terms were “tube position”, “tube placement”, “endotracheal tube”, “ETT”, “endotracheal intubation”, “ETI”, “confirmation”, “verification”, “prehospital”, “out-of-hospital”, and “emergency”. In addition, the reference lists of the studies identified from the database search were reviewed manually to identify any further relevant articles to be included in this review.

**Fig. 1:**
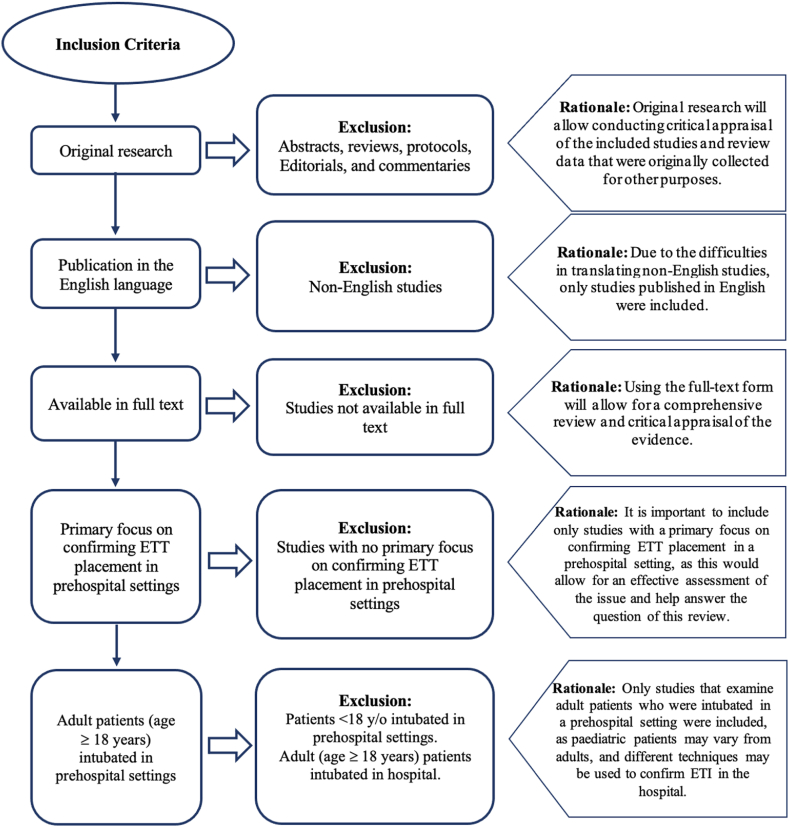
Inclusion and exclusion criteria.

Clear inclusion and exclusion criteria were defined before performing the literature search (Fig. 1). Included studies were limited to a specific time. Any article that was published in full text between June 1, 1992 and June 12, 2022, which is the date of the last database search, and met the determined criteria was included in the review. Studies were included only if they involved observational controlled and uncontrolled studies or interventional studies (randomized and non-randomized) that reported the efficacy of any method used to confirm ETT placement in prehospital settings. Studies where full text was not available were excluded from the review, along with those that were not a report of original research, such as an opinion piece.

### 2.2 Article screening, data extraction, and risk of bias assessment

Studies identified through the search strategy were imported into Rayyan software for systematic reviews [6]. Duplicate studies were removed by the software. Two independent reviewers screened studies by title and abstract and then assessed the eligibility of every study by screening their full-text version. Studies that met the criteria were considered for data extraction. A standardized spreadsheet was used to store the collected characteristics information of each included study. In data extraction, the following data were noted author surname, publication year, country of data origin, type of study, sample size, study population, the method used to confirm ETT placement, sensitivity and specificity of the method, and the main results of the study. The “Risk of Bias in Non-randomized Studies of Interventions” (ROBINS-I) tool was used by two independent reviewers to assess the risk of bias in the included studies [7]. This risk of bias tool assesses seven domains of bias. An overall risk of bias assessment for each included study was determined after summarizing the judgements within each domain. If at least one domain was judged by two reviewers to be at serious or critical risk of bias, then the overall study was considered at serious or critical risk of bias. In case of any disagreement between the two reviewers at any stage of this review, it was resolved through discussions between the two reviewers.

### 2.3 Synthesis and analysis

The findings of included studies were collected reviewed in EXCEL sheet. Due to the heterogeneity of the included studies for each confirmatory methods for ETT placement and/or lack of evidence (for example, only one study investigated the use point of care ultrasound as a confirmatory method for prehospital ETT placement), a thematic literature review using tables with narrative description was determined by two reviewers to be suitable to present the findings of the included studies in this review. Possible reasons for such heterogeneity included the low number of studies addressing each confirmatory method, which may lead to use of different analyses and outcome measures in the available studies aiming to cover the gap in the literature about this topic. After collecting and reviewing the findings from the included studies, they were divided according to the confirmatory methods they assessed. Tables with summary of the findings from the included studies for each confirmatory method was used to allow for descriptive and comparative review.

## 3 Results

### 3.1 Literature search

**Fig. 2:**
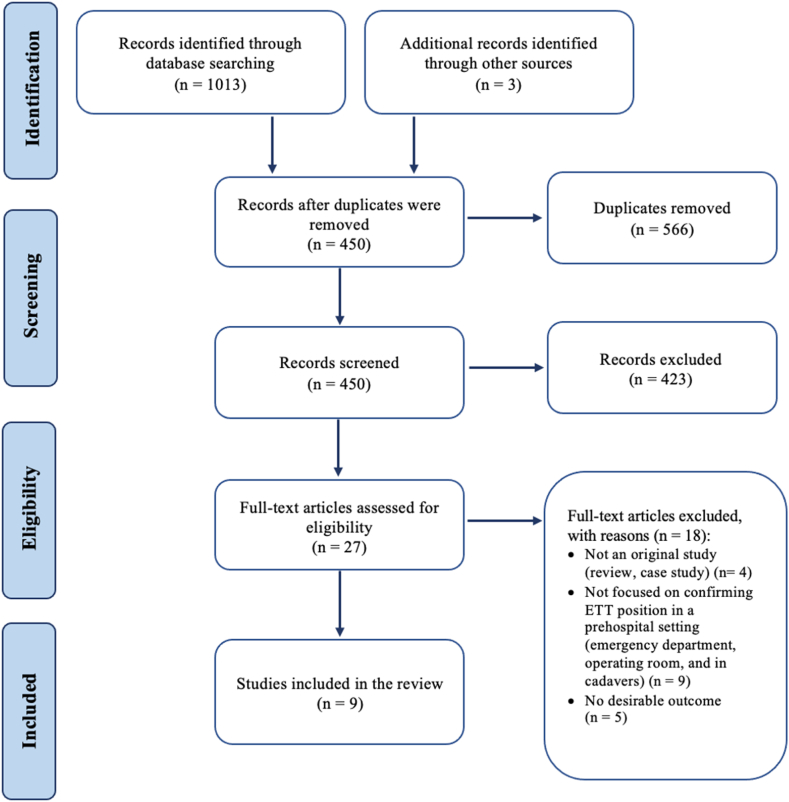
PRISMA flow diagram of the systematic review.

A total of 1016 articles were identified in this review (1013 from database search and three additional articles from the reference list of the included articles). After removing duplicates, 450 citations remained for title and abstract screening. After that stage, 27 articles progressed to full-text reading for potential inclusion in this review. Of these, 18 articles were excluded as they did not our inclusion criteria: not an original study (i.e., review and case study) [[8], [9], [10], [11]], no focus on confirming ETT position in a prehospital setting (i.e., emergency department, operating room, and in cadavers) [[12], [13], [14], [15], [16], [17], [18], [19]], and studies with different outcomes (i.e., not focusing on reporting the accuracy of specific tools) [[20], [21], [22], [23], [24]]. The remaining nine studies met the inclusion criteria and were finally included in this systematic review [[25], [26], [27], [28], [29], [30], [31], [32], [33]]. The process of articles identification, screening, eligibility, and inclusion was reported using PRISMA flow diagram (Fig. 2). The characteristics of the included articles are described in Table 1.

**Table 1:** Characteristics of the included studies.

Author Surname	Publication Year	Country of Data Origin	Study Type	Data Collection Period	Sample Size	Study Population	Method Used to Confirm ETT Placement
S. Zadel et al.	2015	Slovenia	Single-centred prospective study	3 years (January 2011 to January 2014)	124 patients	All orotracheal intubated patients in an out-of-hospital setting regardless of the indication for orotracheal intubation	POCUS, capnography, and auscultation
L. Díaz Díez-Picazo et al.	2010	Madrid, Spain	Preliminary study (prospective study)	1 year (January 2008 to January 2009)	30 patients	Adult patients with non-traumatic cardiopulmonary resuscitation (CPR) who were intubated in a prehospital setting	Capnography
S. Grmec and S. Mally	2004	Maribor, Slovenia	Prospective study	4 years (March 1998 to March 2002)	81 patients	Adult patients with “severe head injury or maxillofacial injury with a need of airway protection or polytrauma” who were intubated by emergency physicians in a prehospital setting	Auscultation, capnometry, and capnography
G. Hendey et al.	2002	California, United States	Prospective, observational study	21 months	53 patients	Intubated patients in the helicopter, at the scene of a prehospital call, or in the originating hospital for interfacility transfers.	Oesophageal detector bulb (ODB)
S. Grmec	2002	Maribor, Slovenia	Prospective study	3 years (February 1998 to February 2001)	345 patients	All adult patients (>18 years) who were intubated by an emergency physician in the prehospital field	Auscultation, capnometry, and capnography

Author Surname	Publication Year	Country of Data Origin	Study Type	Data Collection Period	Sample Size	Study Population	Method Used to Confirm ETT Placement
M. Pelucio et al.	1997	Washington, DC, United States	Prospective, observational study	8 months (October 1993 to May 1994)	374 intubations (ODD used in 213 patients)	All patients (≥18 years old) requiring intubation for medical or traumatic reasons	Syringe ODD
R. Schaller et al.	1997	Virginia, United States	Prospective study	6 months (July 1993 to December 1993)	49 patients	All patients (older than 18 years) who were intubated by the EMS personnel	Colorimetric ETCO_2_ detector compared with an ODD
C.D. Marley et al.	1996	Multiple cities in the United States	Prospective convenience sample in a prehospital setting.	Not reported	92 patients	Intubated adult patients in a prehospital setting	ODD
S R Hayden et al.	1995	Suffolk, United States	Prospective, observational study	2 years and 6 months (December 1990 to May 1993)	566 patients	All out-of-hospital cardiac arrest patients who were intubated in a prehospital setting.	Colorimetric ETCO_2_ detector

#### 3.1.1 Risk of bias within studies

The risk of bias assessment of the included articles is summarized in Table 2. The overall risk of bias was judged to be low in four articles, moderate in one article, and serious in four articles. Articles with a serious risk of bias were published before 2000 due to serious risk bias in confounding variables [30,31] and measurement of outcomes [32,33].

**Table 2:**
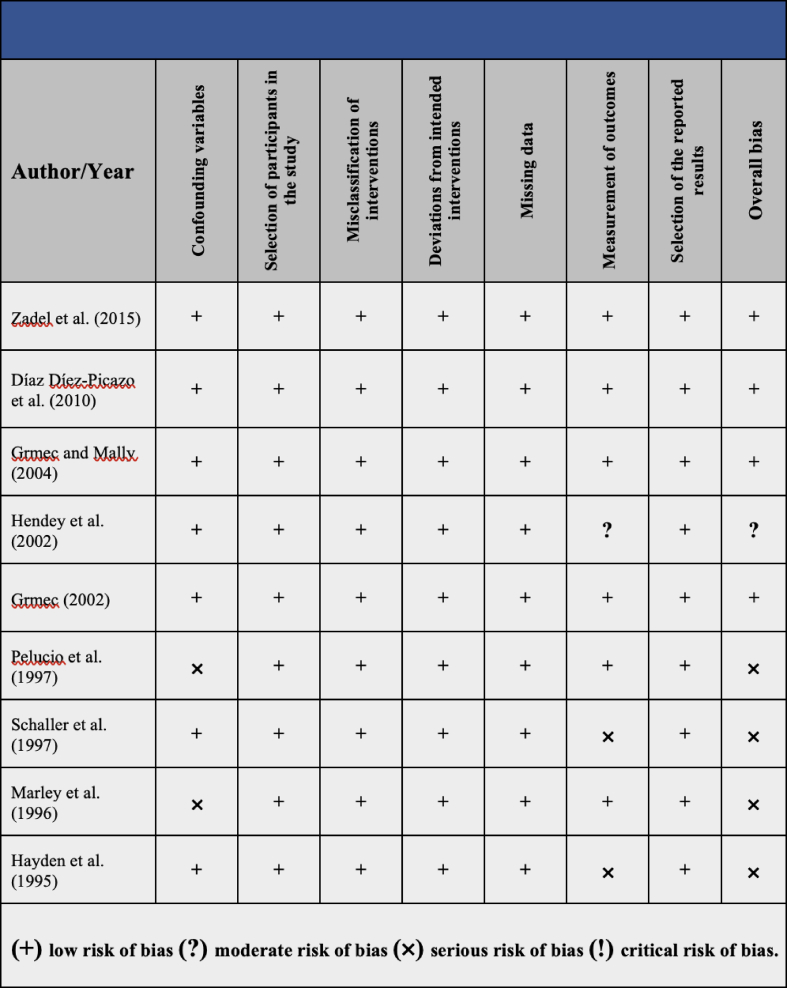
Risk of bias (ROBINA-I) of the included studies.

### 3.2 Data syntheses

The data reported from the included studies were described into different groups according to the confirmatory methods to report the accuracy and the time taken by each method to confirm ETT position in prehospital care: capnography, capnometry, colorimetric ETCO_2_ detector, ultrasound (US), auscultation, and oesophageal detector device (ODD). Also, the limitations associated with any method were identified and reported to overall assess the sensitivity and specificity.

## 4 Accuracy of method used to confirm ETI

• Capnography

In this review, four studies evaluated the accuracy of using capnography to confirm ETT placement in prehospital care [[25], [26], [27], [28]]. In three of them, capnography was compared with other methods such as capnometry, auscultation, and POCUS, while the remaining study by Díaz Díez-Picazo et al. focused on analysing the ability of capnography to confirm ETI with no comparison with other methods.

All four studies showed that the use of capnography was highly accurate and reliable to verify the proper placement of ETT in the prehospital care. Indeed, the sensitivity and specificity of capnography were reported to be 100% in three studies [[25], [26], [27]] with the exception of Díaz Díez-Picazo et al., which highlighted the association of using capnography with 100% positive and negative predictive values [28].

Interestingly, in *Grmec*'s study (2002), capnography was found to be more reliable (100% sensitivity and specificity) in determining the tube position in both arrest and non-arrest patients compared to capnometry and auscultation. The time taken to confirm ETT placement by using capnography was not reported in most of the studies except one, which showed that after initial ETCO_2_ by capnometry 21.4 s (mean) was taken to read the repeated capnography values at seven breaths following ETI insertion [26]). The main findings of the included studies are summarized in Table 3.

• Capnometry

**Table 3:** Summary of the main findings of the included studies.

Study	Sensitivity and Specificity	Main Results of The Study
Zadel et al. (2015)	• Auscultation: 100% sensitivity, 90% specificity • Point of care ultrasound: 100% sensitivity and specificity • Capnography: 100% sensitivity and specificity	• POCUS was reliable, feasible, and accurate method for confirming the tracheal tube placement after urgent intubation with 100% sensitivity and specificity. • POCUS was a more accurate method in distinguishing between endobronchial and endotracheal intubation compared to capnography. • POCUS might be a time-saving method to confirm the proper placement of the tube as the median time needed to perform it was 30 s, with “a large standard deviation from 8 up to 120 s” due to the limitations in out-of-hospital settings such as patient position, space limitation, or weather conditions
Díaz Díez-Picazo et al. (2010)	Not reported	• Capnography effectively confirmed the correct placement of endotracheal tube from the first attempt in 28 out of 30 OHCA patients, while a capnogram was absent in two cases due to oesophageal intubation and appeared when tube was confirmed after the second attempt. • Capnography was associated with 100% positive and negative predictive values
Grmec and Mally (2004)	• Auscultation: 94% sensitivity and 66% specificity • Capnometry: 100% sensitivity and specificity • Capnography: 100% sensitivity and specificity	• Capnometry and capnography were more reliable methods to confirm tube placement in trauma patients compared to auscultation alone. • Combining auscultation with capnometry or capnography is essential to be considered as a reliable method for confirmation of tube placement in trauma patients. • Initial capnometry was the fastest method in determination of tube positioning, “read at the time of tube fixation” and followed by auscultation
Hendey et al. (2002)	ODB: 80% sensitivity and 97% specificity (in the detection of an oesophageal intubation)	• ODB was highly accurate in confirming ETT placement in the aeromedical setting, thus a valuable method. • However, ODB was not able to detect every oesophageal intubation; therefore, its results needed to be carefully interpreted in the context of other methods available to confirm ETT position in a prehospital setting. • Time taken to confirm tube position using ODB was within 5 s in all cases, as per standardized protocol

Study	Sensitivity and Specificity	Main Results of The Study
Grmec (2002)	**In cardiac arrest patients (n = 246):** • Auscultation: 100% sensitivity and 80% specificity • Capnometry: 88% sensitivity and 100% specificity • Capnography: 100% sensitivity and specificity **In non-arrest patients (n = 99):** •Auscultation: 94% sensitivity and 83% specificity •Capnometry: 100% sensitivity and specificity •Capnography: 100% sensitivity and specificity	• Capnography was reliable and accurate in confirming tube position in all arrest and non-arrest patients in prehospital intubations. • Capnometry was able to determine the tube position in all non-arrest patients, whilst incorrect in 28 patients with cardiac arrest: false-negative results in all of these cases (i.e., tracheal tube identified as oesophageal) • In arrest patients, tube position was confirmed to be in proper position using capnometry when an ETCO_2_ value was higher than 5 mmHg at the seventh breath. • Auscultation was a less reliable method in determining proper tube position comparing to capnometry and capnography. • Suggestion that every prehospital team must be equipped with capnography and multiple techniques be routinely used to confirm tracheal tube position in the prehospital setting
Pelucio et al. (1997)	ODD: 50% sensitivity for oesophageal placement and 99% specificity for tracheal placement	• ODD was less sensitive (50%) in detecting oesophageal intubations (5 out of 10 oesophageal intubations) but could confirm 156 out of 158 tracheal intubations. • Confirmation of ETT placement should not solely rely on using ODD as a reliable accurate method in prehospital emergency intubations; other techniques may be required
Schaller et al. (1997)	• ODD: 100% sensitivity and specificity • Colorimetric end-tidal CO_2_ detector: 78% sensitivity, and specificity could not be calculated as there were no oesophageal intubations in this group	• ODD found to be more accurate and sensitive device to confirm the proper placement of ETT compared to the colorimetric ETCO_2_ detection device in the prehospital setting. • ODD might be more precise and easier to use for determination of correct endotracheal tube position than ETCO_2_ devices in prehospital field, particularly in “situations of cardiac arrest requiring CPR”. • Because of the potential for ETT position above the vocal cords in nasotracheal intubation, the ODD results should be cautiously interpreted, and direct visualization with a laryngoscope may be necessary for final confirmation

Study	Sensitivity and Specificity	Main Results of The Study
Marley et al. (1996)	ODD: 100% sensitivity for oesophageal tubes detection and 78% specificity for tracheal tubes detection	• Use of ODD was able to correctly identify all oesophageal intubations in prehospital settings (100%) with no false negatives; however, only 74% of tracheal intubations were correctly identified using ODD. • False positives reported as ODD results in 18 intubations suggested oesophageal placement of the ETT while the tube was correctly placed in the trachea. • In the majority of the intubations, the time required to identify the placement of ETT was less than 5 s. • Combination of methods and good clinical judgment should be used in conjunction with the ODD findings to determine the placement of ETT
Hayden et al. (1995)	Not reported	• In OHCA patients, colorimetric ETCO_2_ detector was reliable method to confirm the correct placement of ETT in 95.6% of arrest patients. • Use of the ETCO_2_ detector should be accompanied with the full clinical assessment to confirm the proper ETT position, as the device might fail to show a “positive colour change” even with the correct tube placement in states of extremely low cardiac output

The use of capnometry to confirm ETI in prehospital care was reported in two studies. One study showed that capnometry was associated with 100% sensitivity and specificity to confirm correct ETT placement in trauma patients with severe head injury [26]. *Grmec* found that capnometry was associated with 100% sensitivity and specificity among non-cardiac arrest patients only, however, capnometry had a false negative result when used for cardiac arrest patients (28 out of 246 ETIs were confirmed by capnometry to be in the oesophagus while it was in the trachea), showing an 88% sensitivity and 100% specificity [25].

The time taken to confirm ETT placement using capnometry was reported in only one study, which showed that capnometry was the fastest method for confirming correct ETT placement compared to auscultation and capnography “at the time of tube fixation”(26). The main findings of these studies are summarized in Table 3.

• Colorimetric End-Tidal CO_2_ Detector

Two studies evaluated the ability of colorimetric end-tidal CO_2_ detector to confirm ETT placement in prehospital care. In Schaller et al.’s (1997) study, colorimetric ETCO_2_ detector was compared with ODDs for verifying correct ETT placement. They found that colorimetric detectors were less accurate in determining correct ETT placement compared to ODD (78% vs. 100% sensitivity, respectively), potentially due to low cardiac output in cardiac arrest patients or severe ventilation-perfusion mismatch. The specificity of colorimetric detectors, however, was not calculated in this study as there were no oesophageal intubations in the patients whose tubes were confirmed using colorimetric detectors [32]. Contrarily, Hayden et al.’s (1995) study demonstrated that in OHCA patients, using a colorimetric ETCO_2_ detector was considered a reliable method to confirm tube placement in most of the intubated arrest patients (541 of 566 patients, 95.6%). Moreover, their findings showed that only one patient out of 541 (0.18%) with a positive colour change of correct tube position was incorrectly intubated into the oesophagus, which was later verified at the receiving hospital. In the remaining 25 patients (4%), colorimetric detectors did not detect any colour changes, and proper placement of the tracheal tube was confirmed through clinical assessment in prehospital care and at the receiving hospital [33]. Collectively, both studies suggested that the use of colorimetric detectors should be accompanied with the clinical assessment to avoid any situations when the device cannot show any colour changes, particularly in patients with extremely low cardiac output states [32,33].

• Auscultation

Three included studies evaluated the accuracy of using auscultation to verify the correct ETT placement. In one study, auscultation was found to be less accurate in confirming proper tube position in non-arrest compared to cardiac arrest patients with a sensitivity and specificity of 94% and 83% compared to 100% and 80%, respectively [25]. In another study, auscultation alone was found to be a less reliable method in confirming ETI with a 94% sensitivity and 66% specificity; instead, it was recommended to be combined with capnography or capnometry to be considered a reliable method [26]. In contrast, *Zadel* et al. (2015) reported 100% sensitivity and 90% specificity associated with the use of auscultation to verify tube placement, demonstrating that auscultation could be used to detect endobronchial tube misplacement. *Grmec and Mally* (2004) reported that the time taken by auscultation for ETT placement confirmation was around 5–10 s after the initial determination with capnometry [26]. Table 3 contains a summary of the main findings of the included studies.

• Point of Care Ultrasound

Over the last 30 years, the use of POCUS to confirm ETT placement in prehospital care was conducted in only one study [27], which aimed to evaluate the accuracy of POCUS in determining ETT position compared to auscultation and capnography. The findings of this study showed that POCUS was a feasible and reliable method for confirming endotracheal intubations with 100% sensitivity and specificity through the assessment of “bilateral lung sliding and diaphragm excursion”(27). In addition, POCUS was more reliable in detecting endobronchial misplacement in three patients in comparison to other methods. Furthermore, it was noticed that the median time required to perform POCUS in prehospital care was 30 s, indicating that POCUS could be used in prehospital care as it is reliable and also time-saving technique (Table 3).

• Oesophageal Detector Devices

The ability of ODD to distinguish between endotracheal and oesophageal intubations was assessed in four studies [[29], [30], [31], [32]]. All studies reported the sensitivity and specificity of ODD with regard to the detection of oesophageal intubation (Table 3). Marley et al. (1996) found that ODD was correct in detecting all oesophageal intubations with 100% sensitivity and 78% specificity for ETT detection, as the device was associated with false positive results in 18 intubations, i.e., tracheal tube identified as oesophageal. Furthermore, in 1997, two studies were published about the use ODDs in prehospital care. One study showed that ODD was more accurate in confirming the correct tube placement (100% sensitivity and specificity) compared to the colorimetric detectors (78% sensitivity, and specificity was not calculated as there were no oesophageal intubations in this group) [32]. However, Pelucio et al.’s study found that using ODD was less sensitive in detecting oesophageal intubations (50% sensitivity, 71% positive predictive value), although it was able to confirm most endotracheal intubations (99% specificity, 97% negative predictive value) [30]. Moreover, the reasons for not detecting oesophageal intubations were not reported in the study [30]. In 2002, Hendey et al. evaluated the accuracy of a bulb-type ODD in the aeromedical setting. They reported that ODD was an accurate method in confirming ETI, with an overall accuracy of 96% and sensitivity and specificity of 80% and 97%, respectively, in detecting oesophageal intubations [29]. Collectively, these studies concluded that the results of ODD should be carefully interpreted and always combined with other confirmation methods as well as good clinical judgment to properly determine tube position.

## 5 Discussion

This systematic review aimed to evaluate the relevant literature about the accuracy of different methods used to confirm endotracheal tubes that were placed by prehospital clinicians. In the majority of the included studies, the findings showed high sensitivity and specificity associated with the methods used to confirm ETT position. Furthermore, capnography was noted to be the most reliable and accurate tool with 100% sensitivity and specificity in all included studies that were conducted to evaluate the use of capnography in comparison to other confirmatory methods [[25], [26], [27], [28]]. Using POCUS as a confirmatory method in prehospital care was also shown to be faster and more accurate than capnography in distinguishing between endotracheal intubations and oesophageal/endobronchial misplacements [27]. However, this statement is supported by the findings of one study only and further studies, therefore, are needed. The remaining methods were associated with different accuracy to confirm ETT placement due to various variables such as the condition of the patient, provider's experience, and prehospital environments, e.g., space limitation, and weather conditions, as explained in Table .3 [[29], [30], [31], [32], [33]]. Therefore, a multi modal approach that encompasses more than one confirmatory method, instead of relying on a singular method, is suggested to improve the overall accuracy of determining the correct tube position in prehospital care.

Cardiac arrest scenarios may impair the accuracy of end tidal capnography as a sole method for ETT confirmation [30,33]. This is another patient encounter which substantiates the need for a multi model approach. However, the use of ETCO2 for cardiac arrest cases are important to provide valuable physiological information as it was shown to be a significant predictor of return of spontaneous circulation (p-value = 0.005) [34] and could be add a great value to the decision-making process to terminate resuscitation [35].

Capnometry was also shown to be less sensitive (88%) for cardiac arrest cases with a specificity of 100%, compared to non-cardiac arrest cases 100% sensitivity and specificity [25]. *Grmec* mentioned that possible reasons for the reported false negative results for cardiac arrest cases included severe ventilation-perfusion mismatch or low cardiac output during cardiac arrest and CPR attempt, which led to low expired CO_2_ concentrations. Therefore, it was suggested that in cardiac arrest patients, an ETCO_2_ value higher than 5 mmHg at the seventh breath or a rise in the level of CO_2_ with each ventilation cycle should be used as a sign to confirm proper tube position when using capnometry [25].

The use of other methods that are not dependent on CO_2_ levels or cardiac output may be more accurate and reliable in patients with low perfusion states [29,32]. Several studies have been conducted to investigate the ability of different methods to determine the proper position of ETT in patients with poor perfusion due to cardiac arrest or shock. In the guidelines from the American Heart Association and the European Resuscitation Council, it is recommended to use waveform capnography as the gold standard to verify the correct ETT position during cardiopulmonary resuscitation [36,37].

In this review, it was found that capnography was the most accurate method in detecting the correct ETT position in both cardiac arrest and non-cardiac arrest patients when compared with other confirmatory methods (as shown in Table 3) [25]. Therefore, it was strongly recommended that prehospital care personnel should be equipped with capnography as a reliable method for ETT placement [25]. Prehospital capnography is mainly used to confirm ETT placements and to help in identifying other respiratory-related emergencies such as asthma, chronic obstructive pulmonary disease, pulmonary embolism, and congestive heart failure [[38], [39], [40]]. As the role of prehospital care personnel is expanding to meet the needs of patients and improve their outcomes, so as the use of assisting technologies including capnography. One example of the future applications of capnography in prehospital care is when Continuous Positive Airway Pressure (CPAP) is applied for patients in prehospital care. Recent evidence showed that early use of CPAP could improve patients and reduce the risk for intubation [41]. Therefore, using capnography in such cases is important to determine the effectiveness of prehospital CPAP [42,43]. However, it should be used with cautious with such cases it is not compatible. The available cardiac monitors with built-in side stream capnography sensors in prehospital care are used to monitor capnography for patients who are breathing spontaneously (through nasal-oral cannula sampler) or those who are intubated (through in-line sampler) [42,44].

In addition, the included studies in this review showed varying sensitivity and specificity rates regarding the use of ODD in prehospital care. For example, the use of ODD in these patients was assessed in Hendey et al.’s study, which found an accuracy of 83% associated with bulb-type ODD in confirming the correct intubations [29]. These findings along with the recommended 30 s wait to make a decision of accurate tube placement make the use of ODD less valuable and inferior to other confirmatory methods of ETT placement. Such device has also less physiologically directed feedback and, therefore, the included studies in the review highlighted that the use of ODD should be always accompanied with other confirmatory methods of ETT placement. Therefore, further studies on the methods that are not dependent on CO_2_ levels, e.g., POCUS, are required to evaluate the reliability of these methods to confirm ETI in different populations in prehospital settings, including cardiac arrest patients.

Accidental ETT misplacement, either oesophageal or endobronchial intubations, as well as accidental dislodgement during patient movement/handover are serious phenomenon that may occur during emergency intubations in prehospital care. Highly accurate confirmatory methods can aid in detecting the incidence of such misplacements [45]. Although several previous studies have analysed multiple methods for ETT position assessment, there is no single method that has proven to be 100% effective in differentiating between endotracheal intubation and oesophageal or endobronchial intubations on its own [25,27,[30], [31], [32]]. Nevertheless, in this review, POCUS was found to be more effective in identifying oesophageal and endobronchial intubations when compared to capnography and auscultation [27]. Such misplacement can be devastating for critically ill/injured patients as it could cause one lung ventilation, which eventually can lead to lung injury [46]. The use of POCUS as a confirmatory method before and after the correction of the ETT from the right main bronchus was shown in this review to be more accurate compared to auscultation and capnography [27]. This finding is consistent with other studies which have shown that the use of POCUS was proven to be an accurate and reliable method for the confirmation of the proper ETT placement in in-hospital airway management conditions [[46], [47], [48]]. With regards to oesophageal intubations, the use of either POCUS or capnography was associated with high accuracy rates [27]. The use of ODD to identify oesophageal intubation, however, showed varied accuracy rates [[29], [30], [31], [32]], although previous literature suggests high accuracy rates associated with the use of ODD [49]. Furthermore, accidental dislodgement of ETT during transportation of patients in prehospital care can lead to serious adverse events if not detected and corrected early. Dislodgement of ETT may occur due to different reasons such as road conditions, patient movement, tube obstruction or dislocation, or system disconnection. Interestingly, waveform capnography in such cases provides an immediate and continuous physiological feedback of patient's status which can prompt early recognition and response of ETT dislodgment [26,50].

Time is a crucial factor in the airway management of critically ill patients. In this review, some studies reported the time taken by the confirmatory method to determine the correct tube position in prehospital care. According to *Grmec and Mally*, capnometry was the fastest method to verify tube placement at the time of ETT fixation when compared to auscultation and capnography. However, this method has been shown to be less reliable in patients with low perfusion states, which is the common indication for prehospital intubations [26]. Hendey et al. (1996) and Marley et al. (2002) were able to determine tube position using ODD in a maximum assessment time of 5 s in the majority of the cases. Although in some studies, it was recommended to wait up to 30 s before determining tube position with ODD, this period of time is not acceptable in prehospital care because of the probability of extending the apnea time in critically ill hypoxic patients which, as mentioned early, makes the use of this confirmatory method inferior to other methods [29,51]. In other methods such as POCUS, a 30-s period may be accepted, since the patients can still be ventilated while assessing the tube position.

As of yet, there is no single method that is completely effective and reliable for the confirmation of ETT position in prehospital care. In this review, multiple methods were used to verify ETT placement, of which all were found to be associated with some potential limitations (Table .4). These methods include capnography, capnometry, auscultation, ODD, and colorimetric ETCO_2_ detectors. Interestingly, some limitations can be significantly critical to a patient's life, which may eventually lead to serious events. For example, failing to detect accidental oesophageal or endobronchial intubations may cause lung injury or even death if it is not detected early [27,46]. Therefore, because of these limitations, simultaneous use of multiple independent confirmatory methods can improve the overall accuracy for the determination of proper tube placement and provide opportunity to identify any accidental ETT misplacements in prehospital care.

**Table 4:** Limitations associated with each method.

Study	Method	Limitation noticed during study:
Zadel et al. (2015)	Capnography	• Capnography failed to distinguish between endotracheal and endobronchial intubation comparing to POCUS and auscultation (i.e., capnography results were normal before and after the correction of orotracheal tube placement)
Grmec (2002)	Auscultation	The use of auscultation to confirm tube position was dependent on the examiner's experience: • In the false positive result in non-arrest patients, the air passing through the oesophagus was incorrectly identified as breath sounds. • However, in false-negative results, emergency physicians were not able to hear the breath sounds clearly due to obesity or incorrect description of breath sounds as stomach gurgling in some conditions such as aspiration, excessive secretions, or pulmonary oedema.
Pelucio et al. (1997) and Marley et al. (1996)	ODD	• ODD may show false ‘normal’ test results, i.e., correct tracheal intubation while tube in the oesophagus, if the ETT cuff was not inflating properly as “the seal provided by the cuff may be necessary to create adequate oesophageal collapse and thereby resistance when the syringe is pulled back”. • Moreover, ODD may show false ‘abnormal’ results and not recognize tracheal intubation if there are any foreign materials that occluded the tracheal tube, such as pulmonary oedema, blood, or vomitus, or if the tube is deeply intubated in the mainstem bronchus.
Schaller et al. (1997)	Colorimetric ETCO_2_ detector	• It was noticed that in cardiac arrest patients, the sensitivity of ETCO_2_ detection device was lower for the assessment of correct tube position, comparing to ODD. • The CO_2_ detector device failed to change color and remained purple, i.e., oesophageal intubation; while the ETT was correctly in the trachea in four arrest patients, and therefore, it was associated with prolonged time of using the device for the final decision making.
Hayden et al. (1995)	Colorimetric ETCO_2_ detector	• Colorimetric ETCO_2_ detector failed to record color changes in some patients when the device became contaminated with medications, vomitus, or secretions. • Moreover, it failed to register any color changes in 25 patients with cardiac arrest although the tube was verified clinically to be in the right position in all patients.

## 6 Limitations

### 6.1 Limitation related to the systematic review

This systematic review is the first review to assess the accuracy of different methods used to confirm the proper ETT placement of in prehospital care. However, there were a number of limitations to this review that need to be highlighted. First, only one study was conducted in the last 10 years and five out the nine included studies were published from 2022 upwards, potentially limiting the applicability of the review findings to current practice. The database search was limited to studies published in English language, which may impact the findings of this review. Furthermore, the sample size was relatively small in most of the included studies, particularly for the cases of most interest, i.e., cardiac arrest and oesophageal and endobronchial intubations.

### 6.2 Limitation related to the nature of the included studies

The nature of the included studies could play a significant role in determining their findings. Different prehospital care system designs could impact the findings of each included study. The level of experience and training of the prehospital care personnel may contribute to the incidence of endotracheal tube misplacement and affect observations about ETT confirmation and accuracy. Moreover, patient physiology and clinical context factor could play a significant role into the reliability of any device (low flow states and capnography).

### 6.3 Limitations associated with each method

Six of the nine included studies reported some limitations associated with the use of different methods to confirm the proper placement of ETT in prehospital care, as shown in Table 4 [25,27,[30], [31], [32], [33]]. In Zadel et al.’s (2015) study, it was noticed that capnography could not distinguish between endobronchial and endotracheal intubations in three right bronchus intubations, as the capnography waveform was normal before and after the correction of tube position in comparison to POCUS and auscultation. Furthermore, in *Grmec*'s (2002) study, auscultation was found to be dependent on the examiner's experience to verify the proper tube position and to avoid the probability of false positive or false negative results. Moreover, Marley et al. (1996) and Pelucio et al. (1997) found that ODD was associated with false ‘normal’ or false ‘abnormal’ test results in various situations, such as not inflating the ETT cuff properly or occluding the ETT with foreign materials, e.g., blood or vomitus. Lastly, for the colorimetric ETCO_2_ detectors, two studies reported two limitations associated with its use in prehospital care: 1) the ETCOs failed to work properly when the device became contaminated with foreign materials, and 2) the ETCO2 was less sensitive in cardiac arrest patients probably due to the low pulmonary blood flow.

## 7 Conclusions

This literature review aimed to assess the accuracy of different methods used to determine ETT position in the prehospital care. Although multiple methods were found to be accurate in verifying the correct ETT placement, there were some limitations associated with each of these methods that need to be confirmed in studies with a larger sample size. Capnography was found to be an accurate and reliable method in many studies, but it may fail to distinguish between endotracheal and endobronchial intubations. Moreover, POCUS was highly reliable and accurate in assessing the correct position of ETT after urgent intubation in the prehospital care, especially in determining tube misplacement into the oesophagus or right main bronchus. However, more studies are needed to prove this finding. The accuracy of other methods varied across the included studies, and they were associated with different limitations questioning their effectiveness in confirming correct ETT position in some patients. Currently, waveform capnography remains the recommended and most reliable method for monitoring and confirming correct ETT placement in prehospital care, although it has some limitations in patients with low perfusion states. Thus, a combination of clinical assessment and multiple confirmation methods, instead of a singular one, is suggested in order to improve the overall accuracy of confirming correct ETT placement in prehospital care.

## Funding

The authors received no financial support for the research, authorship, and publication of this article.

## Ethics approval

This article does not contain any studies with human participants or animals performed by any of the authors.

## Informed consent

For this type of study, no informed consent is required.

## Data availability statement

The data extracted from included studies in this review are available from the corresponding author upon reasonable request.

## Article summary

1 Why is this topic important?

The management of airway in prehospital care is an essential skill for paramedics including advanced airway techniques such as endotracheal tube (ETT) intubation. Confirming ETT placement is of critical importance, as accidental misplacements may occur in prehospital care and could lead to unfavourable outcomes.

2 What does this review attempt to show?

This study, up to our knowledge, is the first to evaluate the effectiveness of applying different methods such as capnography, capnometry, ultrasound, and auscultation to confirm ETT placement in prehospital care. It shows and describe all applied methods reported in the literature regarding the confirmation of ETT placement in prehospital care and assess the accuracy of each applied methods.

3 What are the key findings?
  a. There is no single method with 100% accuracy in confirming the correct ETT placement and detecting the occurrence of accidental oesophageal or endobronchial misplacements in prehospital care.
  b. POCUS and capnography were shown to be accurate in assessing the correct position of ETT after urgent intubation in the prehospital care, however, capnography was less accurate in distinguishing endotracheal from endobronchial intubations.
  c. The accuracy of other confirmatory methods varied across the included studies, and they were associated with different limitations questioning their effectiveness in confirming correct ETT position in some patients.
4 How is patient care impacted?

The findings of this study identified the most accurate methods for confirming correct ETT placement in prehospital care. It also highlighted and listed the limitations of each confirmatory method of ETT placement. These findings will have a significant impact on patient care through.

1. Reducing the risk of ETT misplacement which could lead to adverse outcomes.
2. Enhancing the quality of prehospital care provided for patients requiring emergency care.

## CRediT authorship contribution statement

**Amani Alenazi:** Writing – original draft. **Abdullah Alshibani:** Writing – review & editing.

## Declaration of competing interest

The authors declare that they have no known competing financial interests or personal relationships that could have appeared to influence the work reported in this paper.
